# The role of community-based health planning and services strategy in involving males in the provision of family planning services: a qualitative study in Southern Ghana

**DOI:** 10.1186/1742-4755-10-36

**Published:** 2013-07-26

**Authors:** Philip Baba Adongo, Placide Tapsoba, James F Phillips, Philip Teg-Nefaah Tabong, Alison Stone, Emmanuel Kuffour, Selina F Esantsi, Patricia Akweongo

**Affiliations:** 1 School of Public Health, University of Ghana, Accra, Ghana; 2 Population Council, Accra, Ghana; 3 Mailman School of Public Health, Columbia University, New York, USA

**Keywords:** Community-based health planning and services, Family planning, Male involvement, Reproductive health, Contraceptive use, Spousal communication, Southern Ghana

## Abstract

**Background:**

Reproductive health and Family Planning (FP) services have been of global concern especially in developing countries where fertility rates are high. Traditionally FP services had always targeted females with little or no attention given to males. To ensure equitable distribution of health services, Ministry of Health (MOH), Ghana adopted the Community-Based Health Planning and Services (CHPS) as a nationwide health policy with the aim of reducing obstacles to physical and geographical access to health care delivery including FP services. However, not much is known about the extent to which this policy has contributed to male involvement in FP services. This qualitative descriptive study was therefore designed to explore male involvement in FP services in communities with well functioning CHPS and those with less or no functioning CHPS structures. The study further solicited views of the community on the health status of children.

**Methods:**

This was a qualitative descriptive study and adapted the design of an ongoing study to assess the impact of male involvement in FP referred to as the Navrongo experiment in Northern Ghana. Twelve focus group discussions were held with both male and female community members, six in communities with functional CHPS and six for communities with less/no-functional CHPS. In addition, fifty- nine (59) in-depth interviews were held with Community Health Officers (CHOs), Community Health Volunteers (CHVs) and Health Managers at both the districts and regional levels. The interviews and discussions were tape recorded digitally, transcribed and entered into QSR Nvivo 10^©^ for analysis.

**Results:**

The results revealed a general high perception of an improved health status of children in the last ten years in the communities. These improvements were attributed to immunization of children, exclusive breastfeeding, health education given to mothers on childcare, growth monitoring of children and accessible health care. Despite these achievements in the health of children, participants reported that malnutrition was still rife in the community. The results also revealed that spousal approval was still relevant for women in the use of contraceptives; however, the matrilineal system appears to give more autonomy to women in decision-making. The CHPS strategy was perceived as very helpful with full community participation at all levels of the implementation process. Males were more involved in FP services in communities with functioning CHPS than those without functioning CHPS.

**Conclusion:**

The CHPS strategy has increased access to FP services but spousal consent was very important in the use of FP services. Involving males in reproductive health issues including FP is important to attain reproductive health targets.

## Background

The declaration of Alma-Alta on Primary Health Care (PHC) in 1978 enjoined nations to make health care accessible, affordable, and situated in the cultural context of the people [1]. Following this declaration, nations have employed various strategies to meet the goals of this declaration. As a step to make health care accessible to Ghanaians, in 2005, the Government of Ghana in collaboration with the Ministry of Health and the Ghana Health Services adopted the Community-Based Health Planning and Services (CHPS) as a national policy for the provision of primary health care services [2]. This policy was aimed at reducing obstacles in physical and geographical access to health care delivery to deprived districts and communities in Ghana. The CHPS programme is a scale-up of the “Navrongo experiment” that was initiated and piloted in Northern Ghana over a decade ago. Thus, The Ghana Health Services (GHS) programme of work adopted this model of delivering Primary Health Care (PHC) services as it has the potential of extending health services to poorly served communities in Ghana [3].

The implementation of CHPS at the local level requires the cooperation of the health sector and communities as it involves systematic planning and negotiation with all stakeholders; local authority, political establishment and the community members through community mobilization and effective participation. In this system of health care delivery, the health sector provides additional training on preventive health care services in areas such as immunizations, FP, supervising delivery, antenatal/postnatal care, treatment of minor ailments and health education to community health nurses after which they are relocated to the community to provide door-to-door services. In addition, the communities in consultation with the health sector select community volunteers to support the work of the Community Health Officers (CHOs) in the area of community mobilization and participation, recording vital community statistics and maintaining other essential activities [4]. As a strategy to improve health services delivery, some donor agencies and collaborators, provide assistance to regions to initiate and implement the CHPS strategy. The Population Council through a USAID grant has been in the forefront of implementing CHPS in mainly in the southern sector of Ghana. In Western and Central regions where this study was conducted, Focus Regions and Marie Stopes International have been actively assisting the regions in the implementation of CHPS.

The delivery of FP services is one of the key activities that CHPS strategies emphases. This is a door-to-door strategy of providing health information on FP that targets both women and men. These messages are aimed at changing the perception of men about FP and contraceptive use. This is particularly relevant, as most men in developing countries tend to oppose FP and contraceptive use [5,6]. The patriarchal nature of most African societies often put men in a dominance position over women. This domineering attitude is also transferred across all spheres of life including maternal and reproductive health decisions. Women are expected to kowtow to men when it comes to matters affecting their reproductive rights and their ability to take decisions on contraceptive use and birth control [7,8].

Due to this attitude of men, most FP programmes over the years have viewed men as an obstacle to women’s ability to control their reproductive rights and therefore concentrated on providing the FP programmes to women. FP programmes have focused attention primarily on women, with the intention to free them from excessive childbirth, and to reduce maternal and child mortality through the use of contraceptives. For this reason, FP services were offered within maternal and child health centres, whereas research and information campaigns were tailored to the needs of women with the hope that FP services targeting women will help them to achieve a sustainable socio-economic development and protect their health [9,10]. However, evidence suggests that programme that have involved men and encouraged the use of male methods have led to a drop in fertility and the overall development of women [11]. Men are able to better place their reproductive intentions, promote good decisions for the benefit of their wives when they are made active participants in service delivery programmes.

Socially, contraceptives have been perceived as a liberating strategy for women as it has the potential of providing ability to control fertility. Most men see this as empowering women and a strategy that undermine their authority. In addition, men perceive the use of contraceptives as an opportunity for women to become unfaithful and promiscuous. The desire for large family size, religious considerations, and socio-cultural factors explains the lack of enthusiasm for male involvement in FP programmes [12,13]. However, carefully designed programmes that have used available local male networks to deliver information and activities have proven to reduce social tension thereby improving male involvement in FP activities [6,14].

Inter-spousal communication has been identified as an important consideration in encouraging couples to accept FP and adhere to contraceptive use. Studies have shown that women whose partners approve the use of contraceptives are more likely to use contraceptives as compared to women whose partners’ disapprove contraceptive use [15,16]. This evidence suggests that couples who frequently discuss contraceptive methods are more likely to try other methods when they experience discomfort with a particular contraceptive method. Previous studies also revealed that where both partners approve of the use of contraception, there is high intention and likelihood of future contraceptive use as compared to the likelihood of contraceptive use among partners who object to contraceptive use [17].

Availability and access to FP commodities is very essential in facilitating FP acceptance and use as they are closely linked to the strength and extent of programme coverage. The availability of accurate FP information, quality services and adequate supplies are crucial determinants in the level of male involvement and participation in reproductive health programmes. Low programmes coverage and very poor quality FP services have the tendency to discourage both men and women from FP uptake [18]. The availability of wide range of contraceptive method-mix provides the opportunity for both men and women to make informed choices [19]. Accessible services are a hallmark of high-quality reproductive health programmes. Reliable information, education, communication and counseling provided through primary health care systems have the potential to increase access to the FP clientele. For this reason, the authors decided to investigate the contribution of CHPS, which is now an integral part of Ghana’s primary health care system on male involvement in the delivery and use of FP services.

## Materials and methods

### Ethics statement

Ghana Health Service Ethics Committee approved the protocol for this study. All the respondents in this study provided written or oral informed consent before taking part in this study, which was approved as part of the protocol for the study. To those who gave oral consent, the consent form was translated into the local language by the interviewers. The consenting process was then recorded digitally on a separate digital recorder before the beginning of the interviews and discussions. The digital recorder containing the verbal consents given by respondents were kept separately from the ones used to record the interviews. They were further made to recommend an independent person to serve as a witness and the demographic data of the witness documented. Personal identifiers and locator information were not collected, and any identifying information accidentally mentioned was removed from the text prior to analysis.

### Study area

The study was conducted in Sefwi Bibiani-Ahwiaso Bekwai (SBAB) district and Komenda-Edina-Eguafo-Abrem (KEEA) Municipal area in the Western and Central regions respectively. The 2010 Population and Housing Census put the populations of these two districts at 123, 272 and 144, 705 respectively [20]. The female population for SBAB is 62,417 and that of KEEA at 75,040 [20]. Both SBAB and KEEA are predominantly rural with over 70% of the inhabitants living in areas classified as rural. Both areas are fairly homogeneous with the Akan ethnic group forming about 80% of the population.

These two districts were selected because of an ongoing support the districts were receiving from USAID through Population Council, Ghana to implement CHPS, a community-based health care delivery strategy that has become a national policy in Ghana. Fieldwork took place between June and August 2011 in these two districts. Sefwi-Bibiani-Ahwianso-Bekwai (SBAB) is a district where CHPS implementation is slow (less functioning sites), and Komenda-Edina-Eguafo-Abirem (KEEA) is a district where CHPS has been successfully implemented (well functioning). Atonsu, Ashiem, Behlehem were communities in SBAB that was categorized as areas with less functioning CHPS and were therefore purposely selected for the study. Within the KEEA, two sets of communities were selected, those with functioning and communities with less functioning CHPS. Badukrom, Benyandze, Abeyee, Besease and Aboransa had functioning CHPS whiles Atransa, Bronyibima and Brenu had less functioning CHPS.

### Study design

The study adapted the design of an ongoing study to assess the impact of male involvement in FP on fertility in the “Navrongo experiment” in Northern Ghana. The qualitative descriptive study collected in-depth information on how community-based health care services have been implemented in communities with and without CHPS coverage. The study employed focus group discussions (FGDs) and In-Depth Interviews (IDIs) to collect the data. Semi-structured IDI and FGD guides were designed in English and translated into the local language (Akan) by linguistic experts using a back-to-back translation strategy. Research assistants who were proficient in the local languages were recruited and trained by the researchers. The training covered several hours of classroom work and mock interviews exercises after which they were deployed to the communities for the data collection. Focus group discussions were conducted with 6–8 homogenous community members seated in a semi-circle fashion. Each member of the group was given the opportunity to make their contribution in any question posed before proceeding to another question. Both IDIs and FGDs were completed within 30–60 minutes.

Information gathered under each of the data collection methods included the following:

1. *In-Depth Interviews-* All IDI focused on implementation issues: The extent to which FP is on the agenda of training, deployment strategies, worker selection, refresher training, supervisory encounters, discussion at meetings, community durbars, encounters with village leaders and exchanges with district politicians and administrators.

2. *Focus Group Discussions -* This was to collect normative views from both men and women living in communities with or without CHPS coverage. In all of the focused group interviews, initial questions focused on primary health care and were designed to determine what, if any, reference to FP may spontaneously arise. Interviews then turned to discussions on child health, the current climate in relation to the provision of FP services, perceptions of the importance of these services, and perceptions of social change or reproductive change that may have affected FP demand or service supply. The FGDs were conducted with same discussion guide in all communities.

### Selection of FGD participants

Twelve FGDs were conducted; six in communities with well functioning CHPS and six in communities with less/no CHPS. Interviews were held with both male and female groups in the communities. In the female category, three different groupings were done based on age: 18 to less than 25 years, 25 to 40 years, and more than 40 years. In the males’ category, males below 35 years and those aged 35 years and above groups were formed. Two FGDs were held with the females in each category, one for communities with functioning CHPS and one for communities without functioning CHPS. In the male categories, three FGDs were held for each group equally distributed among communities with functioning and non-functioning CHPS. CHOs and Community Health Volunteers (CHVs) were initial contacted who assisted in recruiting research participants. All participants were married and had between 2–5 children. The age distribution of participants was between 18 years to 59 years. In all 48 females and 42 males took part in the FGDs. Their educational attainments were varied, ranging from elementary to tertiary education.

### Selection of the In-depth interview participants

Fifty-nine IDIs were conducted involving Health Workers, CHVs, CHOs, Public Health Nurses (PHNs), CHPS’ Coordinators at district and regional levels. In all 16 CHOs, 16 CHVs, 20 PHNs, 7 Senior Health Managers took part in the study. The sampling was purposely done in such a way as to ensure equal representation for communities with functioning and non-functioning CHPS. Fourteen of the CHOs were females with only two males. All the PHNs that were interviewed were females. In Ghana, until recently, only females were trained as PHNs because a basic training in midwifery was required to be trained as PHN. The only male public health nurses were those with postgraduate training in public health. Equal numbers of males and female CHVs took part in the study. Three of the Senior Health Managers interviewed were male, and the other three were females.

### Data analysis

All data were audiotaped using digital audio-recorders. Audiotaped data were transcribed into Microsoft Word for Windows. In select cases, the original words or phrases in Akan language were left in the transcripts. The field notes were written on paper and transcribed into the research records. Transcripts were reviewed for obvious errors by both field staff and one of the investigators. Errors were corrected in the transcripts only after discussing the transcriptions with the interviewer/transcriber to ensure appropriate meaning. At least two independent persons read all interviews and “NVivo” coding were conducted to assist in identifying main themes. Nvivo coding involves making written notes on hard copies of the transcripts and reviewing the notes together. From the NVivo coding, a preliminary coding structure was agreed upon and a codebook was created. At that point, all transcripts were entered into NVivo 10^©^, a qualitative software analysis package. Two separate coders conducted the coding.

## Results

### Improvement in child health and the changing perception of reproduction

The study generally revealed an improvement in child health in the last 10 years. These improvements according to respondents were attributable to immunization of children, exclusive breast-feeding, health education given to mothers on childcare, growth monitoring of children and an improvement in access to health care. Both IDIs and FGDs alluded to the fact that immunizations have drastically reduced the incidence of childhood diseases that were fatal in the past. Examples of such diseases were given as measles, poliomyelitis, tuberculosis and whooping cough. Growth monitoring during Child Welfare Clinics (CWCs) also resulted in the early detection of growth challenges and measures taken to ameliorate any adverse effects of such growth challenges. The following responses by respondents support this assertion:

*“In the past children were dying from polio but now there is polio vaccination most of the time. The nurses also go round to inject the children to prevent them from getting the killer diseases. Mothers are also educated on the type of food to give to their kids”-* (A female, FGD in a community with CHPS).

*“First was whooping cough. The children can cough to the extent that all their ribs will be paining them. Second, was a disease that results in skin rashes, which make children skin to develop sores, and at the end of the day the child becomes very weak but now due to the medicines that our CHOs have been giving to the children it is better than the past 10 years”*- (A female, FGD in a community with CHPS).

*“ …. I will say that it (child health) has really improved by God’s Grace; my reason for saying that is because we get help from our health facilities and some medicine they give to our children in the form of immunization like “ntenkyem” (measles)… things have improved better than ten years ago”*- (A male, FGD discussant in a community with CHPS).

Another reasons adduced by respondents as having contributed to the improved health status of children is health education in areas such as nutrition and exclusive breastfeeding. Good nutrition provides the children with nutrients required to build a stronger immunity to fight against diseases. The health benefits of exclusive breastfeeding to both parents and the child were significantly mentioned in both IDIs and FGDs. Exclusive breastfeeding as a contraceptive method (Lactational Amenorrhoea Method) was also stated. The use of mosquito nets as a measure in reducing the incidence of malaria in children was stated as another factor that has contributed to the improved child health.

*“We are advised not to give food or water to our babies for the first six months after birth, and this prevent the children from diarrhea, which in the past was responsible for the death of many children”*- (A mother, FGD in a community with functioning CHPS).

Another factor articulated by respondents as contributing to better child health is an improvement in both geographical and financial access to health care. To respondents, the CHPS system has brought health care to their doorsteps. The doorstep model has resulted in prompt reporting, reducing delays in seeking health.

*“If a child is feverish he/she is immediately brought to the clinic and the CHO takes care, so the mother who previously could not send her child to the “big hospitals” because of money and the distance now bring their children to the clinic to be taken care of. The herbs they previously gave to their children through the instructions of their husbands have also stopped”*- (A CHO, IDI in a community with functioning CHPS).

“*The health of children is now better than before because formally we did not have a clinic here and the nurses were also not coming around to educate us. However, now the nurses come around to educate us on disease prevention”*- (A male, FGD in a community with functioning CHPS).

*“…the CHO and the CHV are very close to us (mothers) in the community, so if you go to them with your problem, they will provide you with the necessary assistance”-* (A mother, FGD in community with functioning CHPS).

The CHPS system has also improved on follow-up of care as the health workers are resident in the community and can easily visit people who report to the clinic for care. This according to respondents was also instrumental in the improvement in child health.

*“If you are sick and you attend the CHPS clinic, the nurses will follow you up to the house to monitor how you are taking your drugs and also ensure that your health is improving. If they (nurses) see that your condition is getting worse, they refer you to the hospital”- (*A woman, FGD in a community with functioning CHPS).

Although the general perception in the community was that, the health of children was relatively better than before as a result of the various interventions initiated by the health sector, malnutrition was still a major problem in the community. Discussants attributed this to high level of poverty in the community.

### The significance of Lineal systems on women’s autonomy

Three main lineal systems are practiced in Ghana: patrilineal, matrilineal and a hybrid of the two. The patrilineal system is mostly practiced among people from the Northern and Volta Regions of Ghana. Matrilineal system is practiced in the middle belt mostly among the Akan-speaking Regions and duo-lineal system is practiced among the people from the Greater Accra Region of Ghana. The study therefore explored the effects of lineal systems on women’s autonomy. It was generally perceived that women in matrilineal systems have better autonomy in reproductive decision-making including the decision to use FP. In the matrilineal system, children born to the male partner in the wedlock are supposed to inherit from the maternal lineage. As a result of this, men appear not to be very much concern about the number of children their wives procreate. Since some men openly state this opinion in public, women in matrilineal systems have often used this practice as a liberating factor to gain some autonomy in reproductive decisions. The upbringing of children in the matrilineal system is more a responsibility of the woman and her lineage. The effect of matrilineal system has been summarized by a statement made by a mother in a FGD.

*“If it had occurred to me earlier, I would have given birth to one or two children because the men in this community do not take care of their children. The men are constantly advised to take care of their nieces and nephews so it makes it difficult for them to take care of their biological children. Twenty (20) out of 100 men take care of their biological children in this community”*- (A mother, FGD in community with less functioning CHPS).

### Social derision and dispute between men and women as a result of the use of Family Planning

Respondents indicated that some community members disrespect and ridicule men and women who do FP. To be able to escape these ridicules, couples are compelled to either do FP in secret or stay away from it.

*“In my community they love to gossip especially about people who are using FP methods, low self esteem is also a challenge since some shy away from accessing the service centres because they would be seen by their neighbours, some hide their cards from their spouse and family to avoid gossips”*- ( A male CHV, IDI, Community with functioning CHPS).

Male respondents in communities without functioning CHPS were firmly of the opinion that endorsing the use of contraceptives was an affront to their authority, as this amounts to giving what a male respondent described as “*power to their wives”*

*“The health workers are trying to give power to the females through the use of FP. Now your wife can tell you that I do not feel like having children anymore….I would marry another wife”*- (A male, FGD in a community with less functioning CHPS).

Another drawback to the use of FP by women was the perception that contraceptive use was associated with promiscuous lifestyle. Men therefore perceived the use of contraceptives as an antidote against unplanned pregnancy that could result from extramarital affairs by women. This was a major issue as majority of the men in these communities are engaged in fishing and it was customary for the men to be away from their homes for several weeks in the sea fishing.

*“Women who use contraceptives become promiscuous because when you go and have sex with another man, there will be no pregnancy. So if you do FP you will be encouraged to have sex with other men when your husband is not around”*- (A man, FGD in community without CHPS).

Three main subthemes emerged in inter-spousal communication on FP. One was an egalitarian discussion, which can result in consensus in either to use or not to use contraceptives. High adherence rates and harmony may be expected in such a situation if couples decide to use. However, in a situation where they both agree not to use, there may be the propensity towards the use of natural method such as having sex only during the safe periods in the woman’s menstrual cycle. This according to respondents has some religious undertones, as some religious sects do not allow their members to use artificial contraceptives.

On the other hand, there may be disagreements in such inter-spousal dialogue on issues regarding contraceptive use. Respondents indicated two scenarios: the man agreeing to the use of contraceptive whiles the woman disagrees or the woman proposing to use contraceptives to the disagreement of the male, each scenario with its ramifications. In a situation where the man disagrees and the woman goes ahead to do FP, according to respondents quarrels have always ensued, which in some instances could result in a break in the marriage. According to respondents, women who use hormonal contraceptives discharge a lot of vagina fluids or become “wet under” which is noticeable by the man as it takes away the pleasure in sex. To female respondents, this could push the man towards engaging in extramarital affairs to get the pleasure the wife is incapable of providing.

*“The reason why it is important to go with your husband to do FP is that, usually when a woman does FP, she easily become wet under, so if the husband have sex with her, he does get the pleasure any longer. This can lead to extramarital affairs by the man and in some instances could lead to divorce because the man does not enjoy sex with the wife any longer”*- (A woman, FGD in a community with functioning CHPS).

If on the other hand the woman refuses to oblige to the husband request to use contraceptives, the man may refuse responsibility for any pregnancy and the child that may result.

*“If your husband asks you to do FP and you refuse and become pregnant, he will deny the pregnancy and neither take care of you nor the child that would be born”*- (A mother, FGD in a community with CHPS).

### Male involvement in family planning

FGDs with women revealed diverse views on the level of male involvement in FP. Some women indicated that their husbands have embraced FP, whiles others stated that their husbands despise FP. Generally, women from communities with functioning CHPS indicated that their men were more accepting of FP than their counterparts from communities without functioning CHPS.

*“My husband has helped me a lot, I have used FP for almost three years and he has even advised that I should go in for a 10 years long method if any”*- (A woman, FGD in community with functioning CHPS).

*“My husband accompanied me to the nurse to listen to the talk on FP and since that time, he has been encouraging me”-* (A woman, FGD in a community with functioning CHPS).

*“I think the men who like FP are now more than those who do not like it. More men are beginning to like FP now because they say that life is difficult, hence many would like to space their children as well as give birth to less children”- *(A man, FGD in a community with CHPS).

Focus group discussions with men in communities with functioning CHPS revealed that men who have knowledge on FP advocate for other men to accept FP. This according to the men is very essential especially in a society where men feel superior and are prejudiced against women.

*“The men who have knowledge on FP are very helpful, just like what teacher was saying about the family of eight children, all living in one room, if you have FP knowledge and you do not educate others on what you know, it is not good. In fact, one family should not have more than three children in one room because when the room is too congested, it can bring about diseases. In my area, those who have knowledge on FP tend to be very helpful, sometimes they bring teachers and midwives/nurses to the churches to give us health education”- *(A man, FGD in a community with functioning CHPS).

*“When I realized that the children were becoming too many, I sat with my wife and had a discussion with her on the need for FP to preserve our strength to take care of the children we already have. This is important so that our children would grow to become better than us. So that was how I supported my wife”- *(A man, FGD in a community with CHPS).

### Male chauvinism and Family Planning use

Male dominance in decision-making affects all aspects of marital life including reproductive issues. Males often prejudice females as inferior and incapable of taking important decisions concerning their life. This emerged as a reason that affects contraceptive use.

*“I think the men think it is all a waste of time, I tell them that making babies is the responsibility of you and your wife, so if you really want to plan your family, it has to be done by both husband and wife. Therefore, even if you come with your wife and she forgets something, you can remind her. However many still refuse to accompany their wives to the clinic”*- (A male CHO in IDI in community with less functioning CHPS).

*“Most of the men think FP is not their responsibility to do, but it is only for women that is why many do not get involved”*- (A woman, FGD in a community without CHPS).

*“The men feel big and see us women as inferior to them, so you cannot tell them what is good for them”*- (A mother, FGD in community with less functioning CHPS).

The study generally revealed that knowledge on contraceptives was universally high amongst males. However, this knowledge has not transformed the thinking of men about women on matters regarding the use of contraceptives or behavioural change. Cultural beliefs and societal perception that FP is “women’s affair”, coupled with various religious beliefs have played a major role in male involvement in FP services. These factors make men feel superior to women, a situation that makes it inappropriate for women to initiate inter-spousal communication on FP.

### Social autonomy, gate keeping and family planning use

Heads of lineage and clans in a typical cohesive family system affect social autonomy and decision-making by members of the family. However, as the extended family system becomes more diffused, leadership, power and authority is increasingly being conferred on husbands who act as heads of nuclear systems. Evidence from this study has shown that husbands still play a major role in the social autonomy of their wives. However, in some instances, where in-laws live with the couple, they can influence all facets of the couple’s life including the decision to use contraceptives. Focus group discussions have revealed that couple whose parents have used contraceptive before either positively or negatively influenced the decision of the couples on contraceptive uptake. Though spousal consent is not required in FP, majority of respondents acknowledged that it was important to seek approval from your partner especially for women because of the benefits in a collective decision as observed by a female respondent:

*“My husband accompanied me to the nurse to listen to the talk on FP and since that time he has been encouraging me to take the pills*”- (A female, FGD in a community with functioning CHPS).

Female respondents attested to the benefits in involving their partners in their FP intentions and use, as men could be very useful when educated to accept FP.

*“My husband reminds me about review dates when I forget and any time we think I am not safe because I have missed a pill, we use a condom*”- (A woman, FGD in a community with functioning CHPS).

### Non-use of Family Planning, pregnancies and abortions

When the discussion turned to the effects of non-use of FP, respondents expressed the view that one of the major consequences in the non-use of contraceptive is an unplanned pregnancy. The study revealed that such unplanned pregnancies ended up been aborted through self-induction using unsafe methods or in private health facilities that perform abortion. FGD discussants stated that women do abortion using traditional and unhygienic methods, which sometimes results in serious complications. Often the women take these drastic actions because they are uncertain of the reaction of family members.

*“…I know a woman who had many children and did not do FP and became pregnant again, she did not want any more children so she wanted to abort the pregnancy and took some medicine and that resulted in her death”- (*A man, FGD in community with less functioning CHPS).

Common drugs mentioned by respondents, as abortificients were cytotec (misoprostol) which is a drug recommended for medical abortion by the World Health Organisation (WHO), *u-pill* and herbal preparations such as *eclandudua, adutwumwaa bitters.* Apparently, these herbal preparations have been licensed by the Food and Drug Authority in Ghana for the management of other diseases but are contraindicated in pregnancy. Adverts for these herbal preparations specifically mentions that women who are pregnant should avoid taking them, and so these drugs have been misconstrued as abortificients and used by community members to induce abortion.

*“Now most of the women when they become pregnant and they do not want to give birth they do abortion. They take in different types of medicines”-* (A man, FGD in a community with less functioning CHPS).

### Community perceptions about Family Planning

Community perception on the use of contraceptives determines their acceptance or rejection of contraceptives and was therefore explored in this study. Three main subthemes emerged regarding community perception from IDIs and FGDs. These were misconceptions, side effects and perceived benefits of contraceptives.

### Misconceptions

The use of contraceptives was associated with various misconceptions that prevented couples especially women from using them. A key misconception that emerged from the respondents was the ability of contraceptives especially intrauterine contraceptive devices (IUCDs) which are inserted into the cervix of a woman to ascend to the heart to cause what respondents described as “uncontrollable heartaches”.

*“The contraceptive that are inserted into the vagina can travel to the heart to give you heartache and other discomfort”*- (A woman, FGD in a community with less functioning CHPS).

Regular menstrual flow (menses) were also perceived to prevent uterine fibroids, hence since the use of hormonal based contraceptives resulted in amenorrhoea (absence of menstrual flow), it was perceived by the community members as predisposing women to uterine fibroids.

*“…I know another lady who did it (FP) and stopped menstruating, later on it developed into fibroid and ended up in surgery”- *(A male participant, FGD in a community with less functioning CHPS).

*“Every drug has its way of working, but my observation is that majority of the clients do not get their monthly menstrual flow due to the mechanism of action of the drugs. The women therefore feel that the lack of monthly menses can cause fibroid or block their tubes and prevent them from having children in future”- (*A male CHO, IDI in a community with less functioning CHPS).

*“My little sister went to do the implant and she changed drastically, that is, first, she was fat but after doing the FP, she grew very lean. Because of what happened, my mother asked her to stop the FP and as soon as she stopped the FP, she gained her weight back” -* (A female, FGD in a community without CHPS).

### Side effects

Just as other medications, hormonal based contraceptives have some adverse reactions when taken by some individuals but this was often over generalized. This was reported as one of factors inhibiting women from using contraceptives. Some of the common side effects that respondents reported were headache, excessive bleeding (menorrhagia), palpitations and dizziness. In principle, not every woman who uses the hormonal contraceptive methods experience these adverse reactions, however the general perception in the community was that all contraceptives except condoms have these side effects.

*“Many also complain about heart and waist problems so many people are scared. In fact, my wife was on FP but she was complaining that anytime she takes the pills she gets palpitations. So she stopped taking the pills and went back after 2 years but still she feels very weak and complains of heartaches, so I have even decided that I would let her give birth to another child after which we would go in for a permanent method through surgery”- *(A man, FGD in a community with functioning CHPS).

Closely related to the side effects is delay in return to fertility following the use of hormonal based contraceptives, which was perceived to be secondary infertility. Respondents generally believed that the use of hormonal based contraceptives could result in secondary infertility.

*“I know someone who has done FP before and after she stopped using it, she is struggling to become pregnant again*”- (A woman, FGD in a community with no CHPS).

Some respondents also indicated that the use of condom takes away the pleasure during sex and therefore not appropriate if one was concerned about the pleasure in sex. Some male respondents when to the extent of suggesting that condom use was not a subject worth discussing between couples.

*“In fact, for the condom we should not talk about it because couples would not enjoy sex when they use the condom. The condom is not nice to use during sex”*- (A man, FGD in a community without CHPS).

### Perceived benefits

There are many inherent benefits in the use of contraceptives to the health and social wellbeing of the couple and the child and this was explored in this study. Respondents that have used or are still using contraceptives stated some of the benefits of using FP methods as helping the couples to space their children, enabling couple to control the number of children they wish to have; making the couple more productive as they have time to concentrate on their work, and improvement in the health of children.

*“FP has helped us to space our children and also provide them with education. It has also improved our productivity since we get time to do our work on time, so it is good*”- (A woman, FGD in a community with CHPS).

*“For me…. I gave birth to two children and became pregnant in less than two years. I later discussed with my husband about FP. He accepted the idea and went to the clinic with me. In the clinic, I was referred to the doctor, who ordered for a test on my blood and urine and he advised me that the injectables would not be good for me but rather I should do the IUD and I did it for 5 years. I actually wanted another boy so I went to remove it and I got pregnant. Even though I was scared that something could happen to me but nothing happen, the only thing is that I put on some small weight, which made me to look very nice and God being so good I had my baby boy. After giving birth to that boy I just told the doctor that I have 5 children so he should ‘cut me’ (sterilize) so I wouldn’t make any more babies again which he did”*- (A woman, FGD in a community with CHPS).

### Barriers to the use of Family Planning

Women reported disapproval by men as a major barrier to the use of contraceptives, which sometimes results in concealed use. However, the perception that women that are using contraceptives discharge too much vagina fluid during sex which is detectable by the husband; it makes it difficult for women to engage in concealed use. Inadequate counseling to make informed choices, correct method use and failure to differentiate between myths and fact were also identified as barrier in the use of contraceptive.

Inadequate method-mix mostly for men emerged as a constrain for men. Male methods covered by the national FP programme are condom and vasectomy. National programmes ignore the traditional methods of withdrawal and periodic abstinence. The result is that men have limited alternatives of the methods of contraception. Another barrier in the use of FP method is the cost of the methods. Even though many respondents stated that contraceptives were affordable, other said contraceptives were expensive and not affordable for them.

*“… you have to pay for the contraceptive but not everybody who want to use them has the money, I met a woman who was interested in doing FP but complained that she did not have money. It is not very expensive but it is not everybody who can afford it; some people do not have money so it becomes a hindrance to accessing the service”*- (A Senior Health Manager in IDI).

Shortages were also cited as a barrier to FP patronage. As some participants stated that sometimes, the health facilities run out of stock of the method of their choice. FP services providers also affirmed this finding. To avert this problem, an electronic stock management system using a short message service on mobile telephone known as the early warning sign system was introduced. In this system, various FP service delivery points are expected to send a text message to their supervising institutions at 2:00GMT every day stating the stock levels of the various FP products, so that prompt supplies could be sent to the facility before they are depleted.

### Sex preferences and ideal age of marriage

There was no consensus on the preferred sex and the age for marriage in the community. FGDs generally indicated that both male and female were a product of God’s blessings and therefore should be acceptable to couples. However, a few respondents did indicate a preference for either a male or female for a child. Reasons cited for preference for the male sex include; their ability to progress to higher educational levels and high school dropout rates for females in the community. The high dropout rates according to community members make any investment in girl child education fruitless. In contrast, respondents who preferred daughters to sons cited reasons such as the propensity of male children to become unruly and indulge in social vices such smoking, stealing that bring ignominy to the family.

*“For me I think girls are better than the boys because some of the boys are “crazy”…. For the boys easily fall prey to peer pressure and indulge in drinking, smoking, and womanizing and at the end of the day nothing good comes out of them”-* (A woman, FGD in a community with CHPS).

Some respondents also buttressed their preference for daughters by referring to a statement made by one of the illustrious sons of Ghana, Dr. Kwegyir Aggrey.

*“Please for me I think giving birth to girls is better than giving birth to boys, because there is a saying that when you educate a man you educate one person but when you educate a woman, you educate the whole nation*”- (A female, FGD in a community with functioning CHPS).

The study further explored the community’s view on the ideal age for marriage. Generally, participants agreed that the ideal age for marriage especially for females was between 20–26 years even though they acknowledged that some woman even marry before celebrating their 18^th^ birthday. This age range according to respondents was ideal because the female would have been physically and emotionally matured enough to face the challenges of pregnancy, childbirth and child rearing. One of the considerations in determining the ideal age for marriage is the ability to cater for the children up to some point before proceeding on the mandatory pension age of 60 years in Ghana. To respondents if one does not marry early and give birth, you were more likely to give birth to what respondents described as “pension babies or pension children”. A pension child is a generic term used to describe children who are still in formal educational institutions whilst their parents have attained the age of 60 years.

“*Please some of the women wait till their late thirties before giving birth, which I think one will give birth to pension babies. Because the woman is already old before giving birth, within the shortest period of time she is on pension”*- (A Female, FGD in a community with functioning CHPS)

### Sources of knowledge on Family Planning

Communication of information and education on FP to both women and men is an important determinant in contraceptive uptake. Awareness of FP methods was universal and, on an average, every participant had heard of contraceptives in the community. The main sources of information on FP methods for men were friends, mass media, non-governmental organisations and health workers. However, male respondents general perceived home-based health education and FP services as more appropriate.

*“The nurses in the community almost always talk about it (contraceptives). If you go to the CHPS compound, she (CHO) talks to you about FP. She also comes to talk about FP using the public address system”-* (A male respondent, FGD in a community with functioning CHPS).

*“When we attend child welfare clinic, the nurses educate us that when we breast feed the children, it contains food and water, so we should do exclusive breast feeding for six (6) months which is also a form of FP for the mother”*- (A female respondent, FGD in a community with functioning CHPS).

*“For us men we prefer health education in our homes…if you come to my house to offer me FP service, nobody in the community will know and I will be comfortable”*- (A man, FGD in a community with less functioning CHPS).

### CHPS and the provision of FP services

The Primary Health Care (PHC) system requires that health delivery system be decentralized and accessible to all individuals irrespective of their geographical location and socio-cultural background. To this end, CHPS was introduced to make health accessible to communities without hospitals, clinics and health posts. The study therefore explored the role of the CHPS in the provision of FP services.

### The role of development partners and NGO in service delivery

Health delivery system involves several stakeholders from both governmental and non-governmental institutions. Effective collaboration between these stakeholders is important in achieving the desire goals in any health care intervention. Several community based non-governmental organisations were mentioned as promoting reproductive health interventions including the use of contraceptives in the study areas. The contributions of these institutions were vivid in the minds of community members and collaborated by the Health Managers in the community.

*“….There are NGOs and agencies like Marie Stopes International helping. Two years ago they were with us and many women came to do the long-term methods”*- (A Health Manager in IDI).

*“Yeah, networking, we use the community volunteers, you know we have some NGOs who are working on the ground, CODISOT is there, they are also doing FP*”- (A Senior Health Manager in IDI).

*“In my church, the nurses are often invited to come and educate us on FP methods and contraceptive use. Now the pastors are preaching that if you do not take care of the children you bring into this world, you will have to give account to God when you die. We have embraced FP in our church and receive information and advice in the church*”- (A woman, FGD in a community with functioning CHPS).

Contrary, some respondents indicated that some religious sects do not allow their followers to use contraceptives. This according to respondents was a drawback to advocacy on the use of contraceptives.

*“We have a church by name…. they worship on Saturdays and preach against contraceptives. They say that a woman should allow the husband to have sex with her anytime he wishes”- (*A woman, FGD in a community without CHPS).

### Implementation related issues

The study further explored the implementation of the CHPS and FP services in the community examining it from the political, community and health sector involvements.

a. **Political involvement**

Respondents stated that they sometimes make appeals to the governmental institutions to provide them with logistics for the CHPS compound and this emerged as a well-entrenched theme in the implementation of the CHPS systems.

*“Some communities request for hospitals but when they are unable to get it, they get the CHPS facility instead. They become intermediaries between the hospital and the community to treat minor ailments”- (*A man, FGD in a community with functioning CHPS).

The District and Municipal Assemblies also assist in the CHPS programme by providing the infrastructure for the programme and accommodation facilities for the health workers. This was however not without challenges. Lack of funding to support the activities of CHVs was given as a major challenge resulting in high attrition rate for CHVs. Funds were also unavailable for refresher training to update the knowledge of both CHVs and CHOs.

*“We are unable to organize periodic training for volunteers because of lack of funding….Even money to motivate the volunteers is a problem. Some of them say they need bicycles to enable them go round the communities which are unavailable”*- (A Senior Health Manager, IDI).

b. **Community involvement**

The CHPS programme is a community-based health intervention strategy. This therefore implies that communities would have to be involved and actively participate at all levels of discussions leading to the establishment of the CHPS compound, recruitment of staff and the management of the CHPS compounds. Respondents generally believed that the communities were involved in the establishment of the CHPS facilities. Participants also stated that FP was an integral part of the activities of the CHOs and CHVs included FP.

*“We had a meeting at our community and discussed it (CHPS) and asked those who were interested and have the desire to work as volunteers to submit their names, so we chose the most knowledgeable amongst them. Whenever a volunteer travels or is no longer interested we choose another person to take over”- (*A man, FGD in a community with functioning CHPS).

*“The communities are doing their best; they provide infrastructure, maybe a room or two to start the CHPS program. About three communities have been here, they want the CHPS, they have seen the importance of CHPS, and they want it in their community by providing us with a facility”*- (A Senior Health Manager, IDI).

The community sometime also assists the health workers that are posted to the health facilities. The communities can even organize durbars and invite the health workers to give them health education or for them to discuss how to improve on the health situation in the community.

*“Our durbars are usually organised by the community leaders and anytime there is a problem and we report to them, they come to our aid”*- (A CHO, IDI in a community with functioning CHPS).

c. **Health sector involvement**

Two main subthemes emerged in the health sector involvement namely; training and deployment of Community Health Officers (CHOs) and the training and deployment of Community Health Volunteers (CHVs).

### ci. Training and deployment of Community Health Officers

Community Health officers are Community Health Nurses who have completed a two-year training programme in an accredited institution and duly registered by the Nurses and Midwives Council of Ghana. They are also given additional training to be officially recognized as CHOs. Hence, all CHOs assigned to CHPS compound have received formal training to manage minor ailments and to refer promptly to the next level, cases above the training. The major challenge was in the area of deployment of the staff. Community members generally believed that they were not often involved in the deployment of staff to the community. The effect of this is that communities sometimes receive people who are not familiar with the local practices and norms within the community.

*“Sometimes they bring people here who cannot speak the language and you have to tell them your problem through an interpreter….This I think is not good enough”*- (A woman, FGD in a community with CHPS).

### cii. Selection, training and deployment of Community Health Volunteers

The study revealed that there were two main ways Community Health Volunteers (CHV) were selected. The communities either selects a person within the community who is deemed desirable for this responsibility or an old CHV could be interviewed by local health managers and deployed to their own community. In each of these recruitment strategies, the community is involved.

*“Yes, it is the community that selects the volunteers. Mostly what we do is we go to the assemblyman, unit committee members and then the chiefs to put the request before them. Generally, they often select a community member who can at least read and write and is devoted to community work. A community member who is well known by community members. The person is then recommended to us to train*”- (A Senior Health Manager, IDI).

*“My colleague and I are interested in health promotion activities in the community, so we were chosen by our community to be trained and we have come back to help the community”- (*A CHV, IDI in a community with functioning CHPS).

Concerning training, all CHV are either formally trained in a structure-training schedule or are sent for mentorship by CHO or to understudy CHV in a CHPS compound.

*“Yes, I have been trained as a volunteer. We come here (CHPS Zone) for training almost every month”*- (A male CHV, IDI in a community with functioning CHPS).

*“We train the CHV for 5 days and organize periodic training for them. Those we are unable to train, we send them to the CHPS compounds to understudy CHOs and trained CHVs”*- (A Senior Health Manager, IDI).

The study probed further to ascertain whether the training of CHV covers contraceptive methods. It was revealed that FP was part of the training of CHV. However, interviews with CHV revealed that their knowledge on FP was low especially regarding adverse reactions.

*“The Ghana Health Service actually train them for a maximum of about two weeks, and the area covered in the training are management of minor ailments, the CHPS concept itself, home visiting, volunteerism, community participation, community mobilization, and family planning”*- (IDI, Senior Health Manager).

## Discussion

The study aimed to explore how CHPS was contributing to efforts in involving males in FP services. The findings suggest that knowledge level of both CHOs and CHVs on FP was minimal, as many could not describe satisfactorily some of the FP methods including their side effects. Given this inadequate knowledge, it will be difficult for such an individual to be able to educate a client very well to disabuse their minds of some of the misconceptions about contraceptives in the community [21,22]. Quality of care is therefore compromised by a lack of trained reproductive healthcare providers and inadequate counseling skills, resulting in clients not receiving accurate information on FP methods or referrals for different methods when they experience side effects. The lack of adequate knowledge among lower level service providers in FP could have fueled the myths on contraceptive use among community members. This issue highlights the need for improved human resource capacity for both the technical and human aspects of reproductive health and FP counseling.

Though respondents indicated various sources of getting information on contraceptives, home-based dissemination of information was the preferred sources, as men who refuse to follow their partners to the health facilities will also have the opportunity to get the information in their homes. This also has an added advantage of identifying other health problems such that health education could be focused on identified health problems. This approach reinforces the need to strengthen the CHPS system and to extend it to all communities. Home visiting therefore should be seen as a very important activity in preventive health as it has the potential of reducing disease burden in deprived communities. Cultural and linguistic competences of the health care providers are an important consideration in the deployment of CHOs to be able to carry out appropriate health education at homes. The linguistic ability or the ability of the CHO to speak the indigenous language should be considered before posting. This may be difficult in heterogeneous societies with different ethnic groups however; it is worth mentioning, as this could be crucial to communicating the right health information to the households and communities.

The CHPS concepts which emphasis a door-to-door health care and FP services was perceived to be very effective in disseminating FP knowledge in the study. FP uptake was high among communities with functioning CHPS than communities without CHPS. This observation was as results of males in communities with functioning CHPS encouraging their partners to do FP. The door-to-door health education and FP services created a platform for men to get involved and to have the opportunity to ask pertinent questions on FP. For this reason, they are able to receive information that is relevant for informed choices. With such knowledge, they also act as FP advocates in the community. Men in this study in communities with functioning CHPS were often more willing to be involved in health interventions but need correct information and communication directed at them. Previous studies report that men have responded positively to health programmes that involved interventions [23-26]. The CHPS concept is therefore an ideal strategy that can ensure effective male involvement in FP as it will ensure home-based access to FP knowledge and provision of services instead of the traditional facility-based FP services, which many men are unwilling to patronize. Studies have shown that it is very difficult for men to have access to accurate, timely and good quality reproductive and sexual health information and services since men are often unwilling to go to services outlets in formal institutions such as hospitals and clinics [27].

Presently in Ghana, the main source of public health financing for community members is the National Health Insurance. There are even community-based initiatives to register the poor and the vulnerable in the community free. However, contraceptives are not included in the list of benefits for registered members and couples who wish to do FP have to pay for the services. The study however revealed that some community members who are willing to do FP are unable to afford the cost of the service making them vulnerable to unplanned pregnancies and its associated consequences. Out of desperation, such women may fall prey to quacks in the community who will prescribe abortificients that may be detrimental to their health. Complications from unsafe abortion often end in the hospital and death as a result of that recorded as maternal deaths increasing the maternal mortality in Ghana. It is important as Ghana accelerate efforts to achieve the MDG 5 (reducing maternal mortality), that contraceptives be added as one of the benefits in the national health insurance. This is not just going to reduce unplanned pregnancies thereby reducing maternal mortality but also has the added advantage of improving child health, which can also propel Ghana towards achieving MDG 4 (improving child health). Studies have showed that children that are born as a result of unplanned pregnancy are more likely to be malnourished with low survival rate [28].

It was also evident that unplanned pregnancies as a result of failure to use contraceptives often end up in abortion using non-orthodox and unsafe methods. Abortion remains a sensitive issue and is general disapproved by the society. In Ghana, abortion is a criminal offence regulated by Act 29, Section 58 of the criminal code of 1960, amended by PNDCL 102 of 1985 [29]. This law permits safe abortion only if the pregnancy is as a result of incest, rape, and cognitive impairment. In addition, when the pregnancy is likely to adversely affect the mental and physical health of either the mother or the foetus. The consequences of such unsafe abortions are complications that can only be managed using advanced medical procedures which are more likely to cost more than the value of financing contraceptives, however, further research is required in this direction. If such a mother dies in the process, it adds to the burden on maternal deaths. Unsafe abortion is the third most common cause of maternal death in Ghana. Unsafe abortion accounts for 11 percent of maternal mortality in Ghana, according to the 2007 Ghana Maternal Health Survey [30]. A trend analysis of the cause of maternal deaths from 2006–2010 at the Tamale Teaching Hospital in Ghana has revealed that unsafe abortions accounted for 11.5% of maternal mortality [31]. It is therefore important for Ghana to re-examine the stand on abortion to make it possible for women who become pregnant accidentally and wish to terminate the pregnancy to have access to safe abortion in the public and private health institutions.

It is also clear from the study that men are the key decision makers in all aspects of reproduction, family formation and health-seeking behaviour despite the fact that in matrilineal communities, the women have some autonomy but this is not without resistance from the men. Apparently, most men are not prepared or do not wish to relinquish their role or share in their authority. Women neither have control over their sexuality nor on their reproductive goals. Educational and advocacy programmes should try to act upon these observations and strengthen intentions of the men who express readiness to share decision-making power with their wives. Any educational programme for influencing reproductive intentions of couples or promotion of contraception, which neglect men, may not have the desired impact. Such men who are willing to share their authorities could be used as role models in educational programmes as such individuals are capable of causing behavioural change in their follow men. Men’s participation is important to the success of FP programmes and to the empowerment of women [32].

The study further revealed that men feel that contraceptive advocacy programmes are highly gendered with less emphasis placed on men. This perception has been goaded by the lack of product mix for males. It also appears that research has overemphasized the vulnerability of women, making men feel superior and creating a sense of self-fulfilling prophesy in women. Though socio-cultural norms may have put men in decision-making positions much to the detriment of women, the scientific world has further fuelled this in research to the extent that many reproductive health researches are targeted at women. This creates a situation where women are supposed to be conventional and obey whatever men say [7]. There should therefore be a paradigm shift with less emphasis on feminism as this has the potential of linking gender related issues to feminism. Training curricular for secondary and tertiary institutions should include issues on reproductive health and gender. This will increase sensitization and inform the youth to view issues with a gender lens. Involving the media in social advocacy and gender sensitization can have far-reaching effects. Hence, the media should be trained and educated on FP since they have the opportunity to reach a wider audience.

The use of electronic stock management system (Daily Short Message Service) to ensure availability of FP deliverables is a finding in this study that is worth emulation by FP service providers. Regular supply and availability of all types of FP deliverables can increase contraceptive prevalence rate. The findings of this study indicate that this innovation has helped in managing contraceptives, ensuring the availability of method-mix for informed choices by couples.

### Limitations of the study

The researchers used independent individuals to do the translations and the translations were verified using back-to-back translation, however, it is possible that some of the statements could have lost their original meanings. Some of the local terms used could not be directly translated to English. To help mitigate this problem, emphasis in analysis was placed on overarching ideas rather than specific word choices or phrasing. In a social interaction like interviews, there may be the tendency for respondents to give socially desirable answers. However, the researchers used both method and data triangulation, that is collecting data on the same subject from both IDIs and FGDs. Both the data collection in IDIs and FGDs were put together to be able to arrive at themes thus ensuring that the final data reflects the situation in the community. In addition, the data were collected to the point of saturation.

## Conclusion

The study revealed a general improvement in child health in the community as a result of preventive health strategies adopted by health workers. The CHPS strategy has increased access to FP services however; there is the need for service providers to equip themselves with in-depth knowledge on various methods and side effects to be able to provide accurate information for informed choices. The door-to-door strategy espoused by CHPS provided men with the opportunity to have access to information on FP to inform behavioural change. It also ensures couple-based counseling on FP unlike the traditional facility-based services that are unable to capture men, as many men are often unwilling to accompany their wives to the clinics. To this end, the level of FP acceptance among men was high in communities with CHPS than those without CHPS. Socio-cultural barriers inhibiting the use of FP was also less prominent in communities with functioning CHPS. However, spousal approval was still very important in FP services. It is therefore important to strengthen advocacy on involving men in FP. Generally, there was a changing perception of men towards the use of contraceptives as many are now willing to support their wives on issues related to reproductive health including FP.

## Competing interests

The authors declare that they have no competing interests.

## Authors’ contributions

Conceived and designed the experiments: PBA, PT, JFP, AS, Performed the experiments: PT, EK, PBA, SFE Analyzed the data: PBA, PA, PTT, PA. Contributed reagents/ materials/analysis tools: PT, SFE, EK, AS, PTT, PBA. Wrote the paper: PBA, PTT, PA, PT, JFP. All authors read and approved the final manuscript.
